# Effects of NB001 and gabapentin on irritable bowel syndrome-induced behavioral anxiety and spontaneous pain

**DOI:** 10.1186/1756-6606-7-47

**Published:** 2014-06-16

**Authors:** Ming-Ming Zhang, Shui-Bing Liu, Tao Chen, Kohei Koga, Ting Zhang, Yun-Qing Li, Min Zhuo

**Affiliations:** 1 Department of Anatomy, Histology, Embryology & K. K. Leung Brain Research Centre, The Fourth Military Medical University, Xian, Shanxi 710032, China; 2 Department of Physiology, Faculty of Medicine, University of Toronto, 1 King’s College Circle, Toronto, Ontario M5S 1A8, Canada; 3 Center for Neuron and Disease, Frontier Institutes of Life Science, and Science and Technology, Xi’an Jiaotong University, 28 Xianning West Road, Xian, Shanxi 710049, China

**Keywords:** Irritable bowel syndrome, Zymosan, Visceral pain, Spontaneous pain, Anxiety

## Abstract

Irritable bowel syndrome (IBS) is characterized by recurrent abdominal discomfort, spontaneous pain, colorectal hypersensitivity and bowel dysfunction. Patients with IBS also suffer from emotional anxiety and depression. However, few animal studies have investigated IBS-induced spontaneous pain and behavioral anxiety. In this study, we assessed spontaneous pain and anxiety behaviors in an adult mouse model of IBS induced by zymosan administration. By using Fos protein as a marker, we found that sensory and emotion related brain regions were activated at day 7 after the treatment with zymosan; these regions include the prefrontal cortex, anterior cingulate cortex, insular cortex and amygdala. Behaviorally, zymosan administration triggered spontaneous pain (decreased spontaneous activities in the open field test) and increased anxiety-like behaviors in three different tests (the open field, elevated plus maze and light/dark box tests). Intraperitoneal injection of NB001, an adenylyl cyclase 1 (AC1) inhibitor, reduced spontaneous pain but had no significant effect on behavioral anxiety. In contrast, gabapentin reduced both spontaneous pain and behavioral anxiety. These results indicate that NB001 and gabapentin may inhibit spontaneous pain and anxiety-like behaviors through different mechanisms.

## Background

Visceral pain occurs after mechanical or chemical stimulation in and around the internal organs. This form of pain is vague and poorly localized [1-4]. Typical phenotypes of visceral pain include hyperalgesia, in which enhanced pain responses are induced by weak noxious visceral stimuli, and allodynia, in which innocuous visceral stimuli can cause pain. In addition, spontaneous pain or discomfort can frequently be observed [5]. Irritable bowel syndrome (IBS), a major form of chronic visceral pain, is accompanied by visceral hyperalgesia, abnormal bowel habits and spontaneous pain or discomfort [6,7]. Recent studies indicate that an enhanced primary sensory afferent derived from the colorectum maintained IBS-related pain and associated colorectal hypersensitivity [7-9]. It is very likely that enhanced sensory inputs into the central nervous system may trigger long-term changes in brain areas associated with emotional and cognitive processing. Unsurprisingly, it has been reported that IBS is accompanied by severe anxiety, stress and depression [10-15]. Most of previous animal studies of IBS only focused on sensory mechanisms; few examined behavioral and emotional changes in IBS.

Cortical and subcortical areas, such as the prefrontal cortex (PFC), anterior cingulate cortex (ACC), insular cortex and amygdala, are important for processing pain, discomfort and emotional responses [16-23]. Animal and human brain imaging studies show that IBS affects neuronal activities in these brain areas, specifically the ACC [24-30]. In previous studies, we found that pain and pain-related emotional changes triggered long-term potentiation (LTP) of synaptic transmission in the ACC [18,31,32]. Calcium-stimulated adenylyl cyclase subtype 1 (AC1) plays important roles in pain-related LTP in the ACC as well as in injury-induced plastic changes [33-36]. Genetic inhibition of AC1 or administration of the AC1 inhibitor NB001 causes analgesic effects in mice with somatic tissue injury, inflammation or muscle pain [37-39]. However, the possible effect of an AC1 inhibitor on IBS-induced pain or anxiety has not been investigated.

In the present study, we began by labeling Fos protein, a marker of neural activity, in mouse brains to detect the activation of brain areas that are important for pain processing as well as the processing of anxiety and fear. Second, we wanted to establish behavioral measurement of spontaneous pain, discomfort and behavioral anxiety in adult mice with intra-colonic injection of zymosan, a common animal model to mimic the clinical IBS [40,41]. Finally, we evaluated the effects of the AC1 inhibitor NB001 and gabapentin, a commonly used anticonvulsant and analgesic component in treating seizures and somatic neuropathic pain [42-44], on spontaneous pain and behavioral anxiety, as well as other behavioral responses.

## Results

### Zymosan increases the expression of Fos protein in the central nervous system

**Figure 1:**
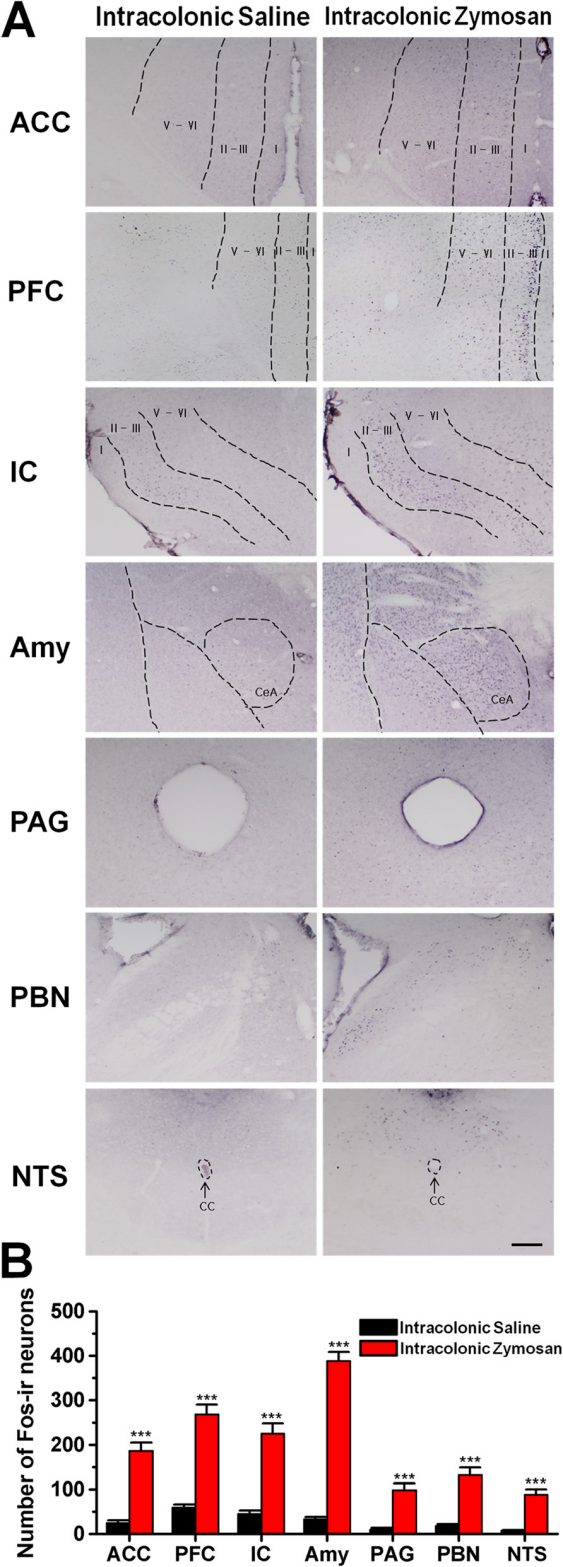
**The expression of Fos protein was upregulated in different brain regions in zymosan-treated mice. (A)** Immunohistochemical staining for Fos in the ACC (anterior cingulate cortex, 0.98 mm prior to bregma), PFC (prefrontal cortex, 1.78 mm prior to bregma), IC (insular cortex, 1.18 mm prior to bregma), Amy (amygdala, 1.46 mm posterior to bregma), PAG (periaqueductal gray, 4.72 mm posterior to bregma), PBN (parabrachial nucleus, 5.20 mm posterior to bregma) and NTS (nucleus of solitary tract, 7.76 mm posterior to bregma) at day 7 after intracolonic injection of zymosan or saline (control). (CeA: central amygdala; cc: central canal; Scale bar: ACC, PFC and IC, 250 μm; Amy and PAG, 150 μm; PBN and NTS, 100 μm). **(B)** Histogram showing the quantification of Fos-immunoreactive (Fos-ir) neurons in the above brain regions of saline- and zymosan-treated mice (n = 6 slices per mouse; 6 mice per group). * denotes a significant difference between zymosan- and saline-treated mice.

First, we sought to determine whether IBS activates sensory and emotion related brain areas. Neuronal Fos protein expression is commonly used as a correlative indicator of neuronal activity induced by somatic or visceral noxious stimuli [20,45,46]. We performed Fos immunohistochemical staining to determine whether cortical and subcortical neurons are involved in zymosan-induced colitis on day 7 after injections of zymosan. This time point was chosen because our preliminary experiments have indicated that the mice display obvious visceral pain behaviors on day 7 after injections of zymosan. Quantification of Fos-immunoreactive (Fos-ir) neurons showed that Fos was expressed at higher levels in many areas of the forebrain, subcortex and brainstem of zymosan-treated mice on day 7 than in the same brain regions of saline-treated mice on day 7 (n = 6 slices from 6 mice per group; Figure 1). In forebrain areas, zymosan treatment induced prominent Fos staining in layer II-III of the ACC (zymosan: 186.4 ± 8.9; saline: 14.9 ± 2.5; unpaired *t*-test, *P* < 0.001), the medial PFC (zymosan: 268.7 ± 11.4; saline: 59.6 ± 5.3; unpaired *t*-test, *P* < 0.001) and layer II-III of the insular cortex (zymosan: 225.4 ± 12.3; saline: 45.2 ± 2.1; unpaired *t*-test, *P* < 0.001). In the subcortex areas, Fos expression was mainly found in the central nucleus of the amygdaloid complex (zymosan: 388.5 ± 10.6; saline: 33 ± 3.3; unpaired *t*-test, *P* < 0.001). In the midbrain, zymosan treatment augmented Fos expression within the ventral posterolateral region of the periaqueductal gray (PAG) (zymosan: 98.3 ± 5.2; saline: 9.5 ± 1.9; unpaired *t*-test, *P* < 0.001). In the brainstem, abundant Fos expression was also observed in the lateral region of the parabrachial nucleus (PBN) (zymosan: 132.5 ± 6.6; saline: 17.4 ± 1.1; unpaired *t*-test, *P* < 0.001) and in the medial and commissural parts of the nucleus of solitary tract (NTS) (zymosan: 87.9 ± 7.1; saline: 7.9 ± 1.0; unpaired *t*-test, *P* < 0.001). These observations suggest that brain regions associated with pain and emotion are activated in response to intracolonic injection of zymosan.

### Zymosan-induced visceral pain behaviors

**Figure 2:**
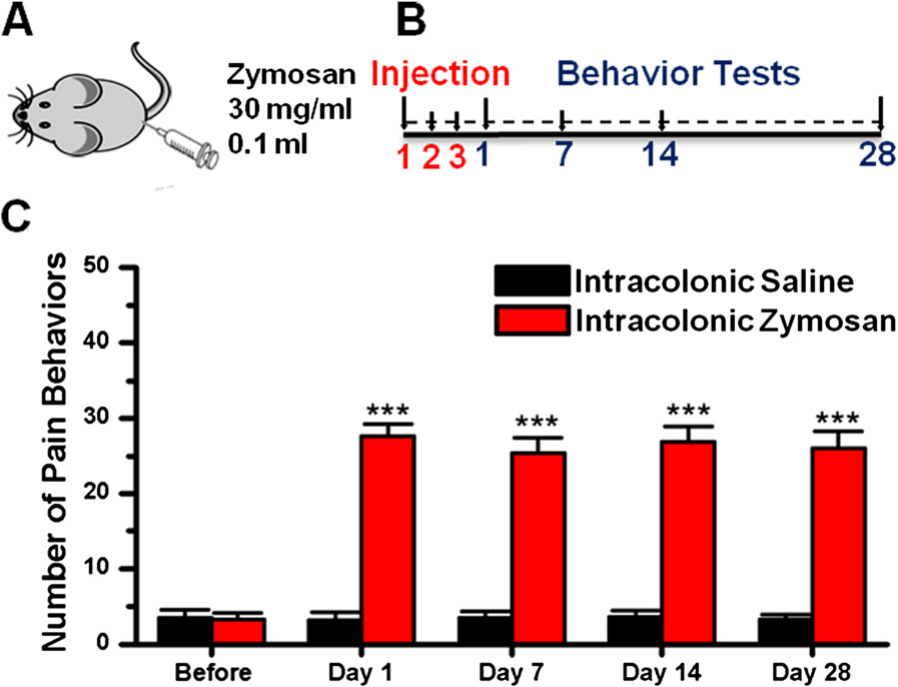
**Behavioral tests of zymosan-treated mice. (A)** A volume of 0.1 ml zymosan (30 mg/ml) or saline was injected transanally into the colons of mice via a needle connected to a PE tube. **(B)** After injections on three consecutive days, behavioral tests were administered to zymosan- and saline-treated mice on days 1, 7, 14 and 28 (n = 6 mice per group). **(C)** The number of pain behaviors observed in zymosan-treated mice on days 1, 7, 14 and 28 increased significantly compared to saline-treated mice (n = 6 mice per group). * denotes a significant difference between zymosan- and saline-treated mice. “Before” designated the mice before saline or zymosan intracolonic treatment.

We showed that brain regions related to pain and emotional processing were activated in zymosan-induced colitis, as shown by Fos immunohistochemical staining. Next, we measured behavioral responses to confirm that visceral pain was induced by intracolonic injection of zymosan in adult mice [41,47,48]. As reported previously, mouse postures defined as visceral pain-related behaviors include licking of the abdomen in the absence of other grooming behavior, whole-body stretching, flattening the abdomen against the floor, or contracting the abdominal wall such that an arched posture is adopted for 1–2 sec (abdominal retractions) [49]. To evaluate the time course of zymosan-induced visceral pain, 12 mice were divided into zymosan- and saline-treated groups (n = 6 mice per group). After intracolonic injections of zymosan or saline for three consecutive days (Figure 2A), mice were then tested for visceral pain behaviors on days 1, 7, 14 and 28 (Figure 2B). We found that zymosan-treated mice exhibited obvious visceral pain-related behaviors on the first day compared to saline-treated mice (zymosan: 27.7 ± 1.6; *vs.* saline: 3.2 ± 1.1; *P* < 0.001; repeated measures ANOVA followed by *post hoc* comparison with LSD test; Figure 2C). Abdominal, visceral pain-related behaviors persisted for a long period of time (zymosan: day 7: 25.3 ± 2.1; day 14: 26.8 ± 2.1; day 28: 26.0 ± 2.2; *vs.* saline: day 7: 3.5 ± 0.9; day 14: 3.7 ± 0.9; day 28: 3.3 ± 0.7; *P* < 0.001; repeated measures ANOVA followed by *post hoc* comparison with LSD test; Figure 2B-C). In zymosan-treated mice, there were no differences in the quantity of pain-related behaviors within time points (*F*_(3, 20)_ = 0.25, *P* = 0.86; one way ANOVA; Figure 2C), suggesting that intracolonic injection of zymosan can induce stable, chronic visceral pain. Thus, our data indicate that intracolonic injection of zymosan can induce obvious and persistent visceral pain behaviors in mice.

### Spontaneous pain increased in zymosan-treated mice

**Figure 3:**
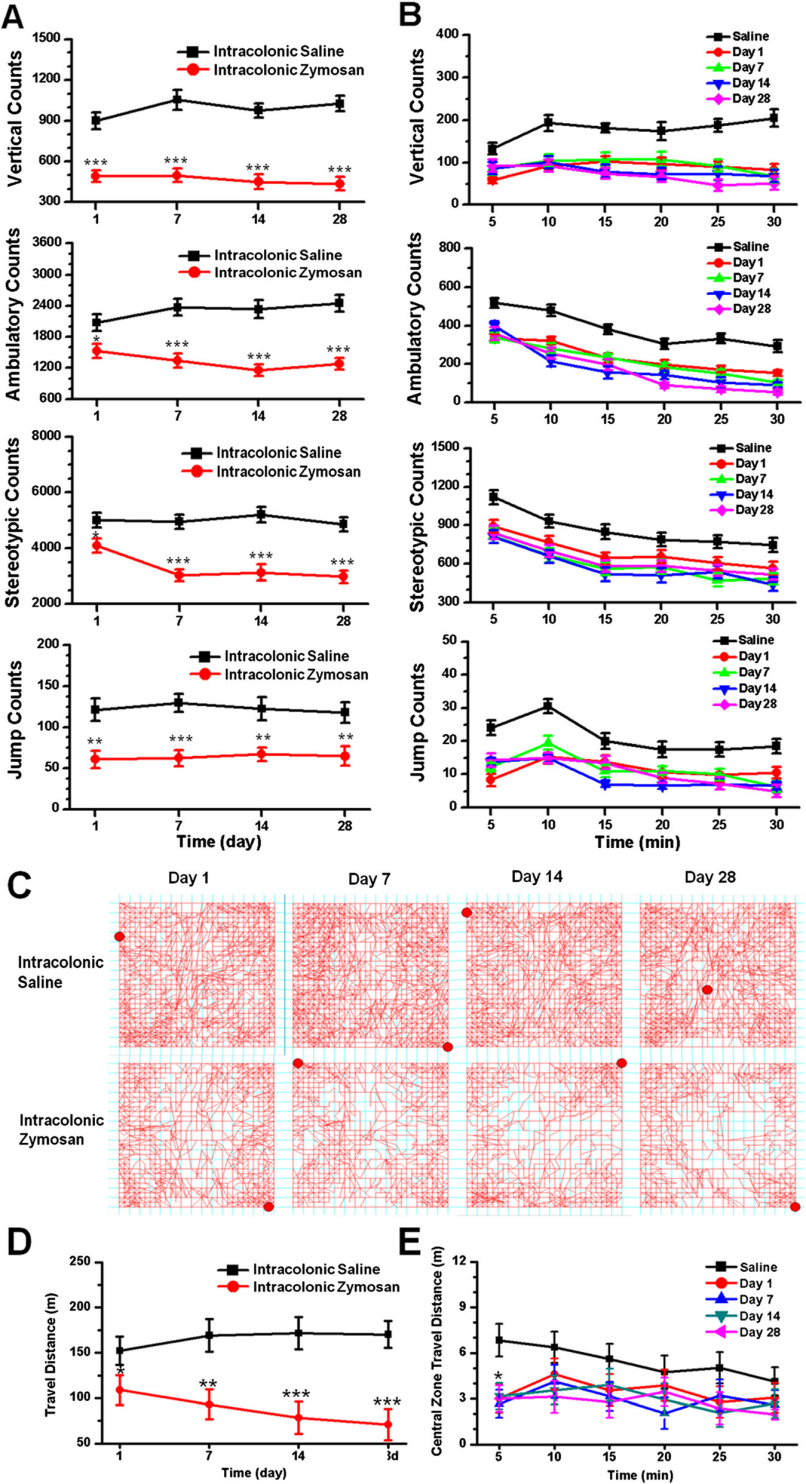
**Behavioral assessment of zymosan-treated mice in the open field. (A)** Counts of vertical, ambulatory, stereotypic and jump behaviors in zymosan-treated mice were decreased compared to saline-injected mice on days 1, 7, 14 and 28 day (n = 6 mice per group for each testing day). * denotes a significant difference between zymosan- and saline-treated mice. **(B)** Vertical, ambulatory, stereotypic and jump counts over 30 min on days 1, 7, 14 and 28 for zymosan-treated mice (n = 6 for each testing day) and saline-treated mice (n = 24 mice). **(C)** Representative traces show the movement of zymosan- and saline-treated mice in the open field test over a period of 30 min. **(D)** Zymosan-treated mice traveled significantly less distance than did saline-treated mice on days 1, 7, 14 and 28 (n = 6 mice per group for each testing day). * denotes a significant difference between zymosan- and saline-treated mice. **(E)** The distance traveled by zymosan-treated mice in the central zone within the first 5 min of the 30-min session of the open field test was significantly reduced on days 1, 7, 14 and 28 (n = 6 mice for each testing day) compared to saline-treated mice (n = 24 mice). * denotes a significant difference from saline-treated mice.

To further explore spontaneous pain of zymosan-treated mice, we carried out experiments using the open field test. To avoid habituation effects, independent groups were used for each testing day. Thus, each mouse was tested only once in the open field. In the open field test, both spontaneous activities and travel distance were measured automatically. Spontaneous activities include vertical (rearing), ambulatory (horizontal activity), stereotypic (absence of locomotion but presence of repetitive, invariant behaviors such as grooming and head bobbing) and jump (escape latency) behaviors. Interestingly, counts for all four of these behaviors were significantly decreased in zymosan-treated mice on day 1 compared to saline-treated groups. These decreases maintained on days 7, 14 and 28 (n = 6 mice per group for each testing day, two-way ANOVA followed by *post hoc* comparison with LSD test; Table 1, Figure 3A). There were no significant differences among the day 1, 7, 14 and 28 groups (Vertical: *F*_(3, 40)_ = 2.57, *P* = 0.07; Ambulatory: *F*_(3, 40)_ = 2.41, *P* = 0.08; Stereotypic: *F*_(3, 40)_ = 2.21, *P* = 0.10; Jump: *F*_(3, 40)_ = 0.14, *P* = 0.94; two-way ANOVA followed by *post hoc* comparison with LSD test; Table 1, Figure 3A). As comparison, there were no significant differences among saline-treated mice (n = 6 mice for each time point) and naïve mice (n = 6 mice). An analysis of our behavioral data with 5-min epochs showed that the counts decreased consistently in zymosan-treated mice within the 30-min testing time period compared to saline-treated mice (Figure 3B).

**Table 1:** Spontaneous activities counts of mice in the open field test

**Counts**	**Day**	**Intracolonic saline**	**Intracolonic zymosan**	***P***
Vertical	1	800.5 ± 63.8	492.2 ± 42.4	< 0.001
	7	1055.3 ± 72.8	498.0 ± 51.8	< 0.001
	14	976.7 ± 50.6	450.5 ± 53.2	< 0.001
	28	1026.8 ± 58.6	438.2 ± 49.9	< 0.001
Ambulatory	1	1979.3 ± 158.2	1404.0 ± 133.3	< 0.05
	7	2374.7 ± 155.7	1343.0 ± 152.5	< 0.001
	14	2336.7 ± 172.2	1155.0 ± 117.4	< 0.001
	28	2448.3 ± 161.9	1279.7 ± 147.4	< 0.001
Stereotypic	1	5011.3 ± 254.8,	4094.3 ± 257.1	< 0.05
	7	4961.3 ± 260.0	3024.8 ± 214.6	< 0.001
	14	5206.7 ± 265.7	3124.8 ± 289.2	< 0.001
	28	4862.7 ± 259.3	2970.2 ± 218.7	< 0.001
Jump	1	120.8 ± 13.9	60.7 ± 10.6	< 0.01
	7	129.5 ± 10.9	62.7 ± 9.7	< 0.001
	14	122.5 ± 14.0	67.0 ± 8.5	< 0.01
	28	117.8 ± 12.3	64.7 ± 11.5	< 0.01

*P*: in comparison with saline intracolonic treated group; n = 6 mice per group.

In addition to four major behavioral counts, the total travel distance of zymosan-treated mice in the open field also markedly decreased days 1 (*F*_(1, 40)_ = 4.83, *P* < 0.05), 7 (*F*_(1, 40)_ = 10.56, *P* < 0.01), 14 (*F*_(1, 40)_ = 15.75, *P* < 0.001) and 28 (*F*_(1, 40)_ = 17.97, *P* < 0.001) (n = 6 mice per group for each testing day, two-way ANOVA followed by *post hoc* comparison with LSD test; Figure 3D). The magnitude of reduction was similar among the day 1, 7, 14 and 28 groups (*F*_(3, 40)_ = 0.83, *P* = 0.49; two-way ANOVA; Figure 3D). These results consistently suggest that behavioral changes in the open field test can be used to predict the magnitude of spontaneous pain.

### Zymosan-induced anxiety-like behaviors

Anxiety-like behavior in the open field is often assessed by measuring exploratory behavior in the central field within the first few minutes of beginning the open field test [37,50]. Therefore, we analyzed the travel distance within the central zone for zymosan-treated groups at different time points, using a 5-min epoch. We found that zymosan-treated mice travelled less distance within the central area within the first 5 min of starting the test compared to saline-treated mice (zymosan: day 1: 3.0 ± 0.9 m, *P* < 0.05; day 7: 2.5 ± 0.9 m, *P* < 0.05; day 14: 3.2 ± 0.8 m, *P* < 0.05; day 28: 3.1 ± 0.8 m, *P* < 0.05; *vs.* saline: 6.9 ± 1.1 m; n = 6 mice per group for each testing day, two-way ANOVA followed by *post hoc* comparison with Dunnett’s test; Figure 3E). The magnitude of reduction was similar among the day 1, 7, 14 and 28 groups (*F*_(3, 20)_ = 0.06, *P* = 0.98; two-way ANOVA; Figure 3E). We also found that zymosan-treated mice spent less time in the central area within the first 5 min of starting the test compared to saline-treated mice (zymosan: day 1: 3.2 ± 0.6 s, *P* < 0.001; day 7: 2.7 ± 0.5 s, *P* < 0.001; day 14: 3.5 ± 0.5 s, *P* < 0.001; day 28: 3.1 ± 0.6 s, *P* < 0.001; *vs.* saline: 8.9 ± 0.9 s; n = 6 mice per group for each testing day, two-way ANOVA followed by *post hoc* comparison with Dunnett’s test). These findings indicate that zymosan-treated mice likely experience more anxiety than saline-treated mice.

**Figure 4:**
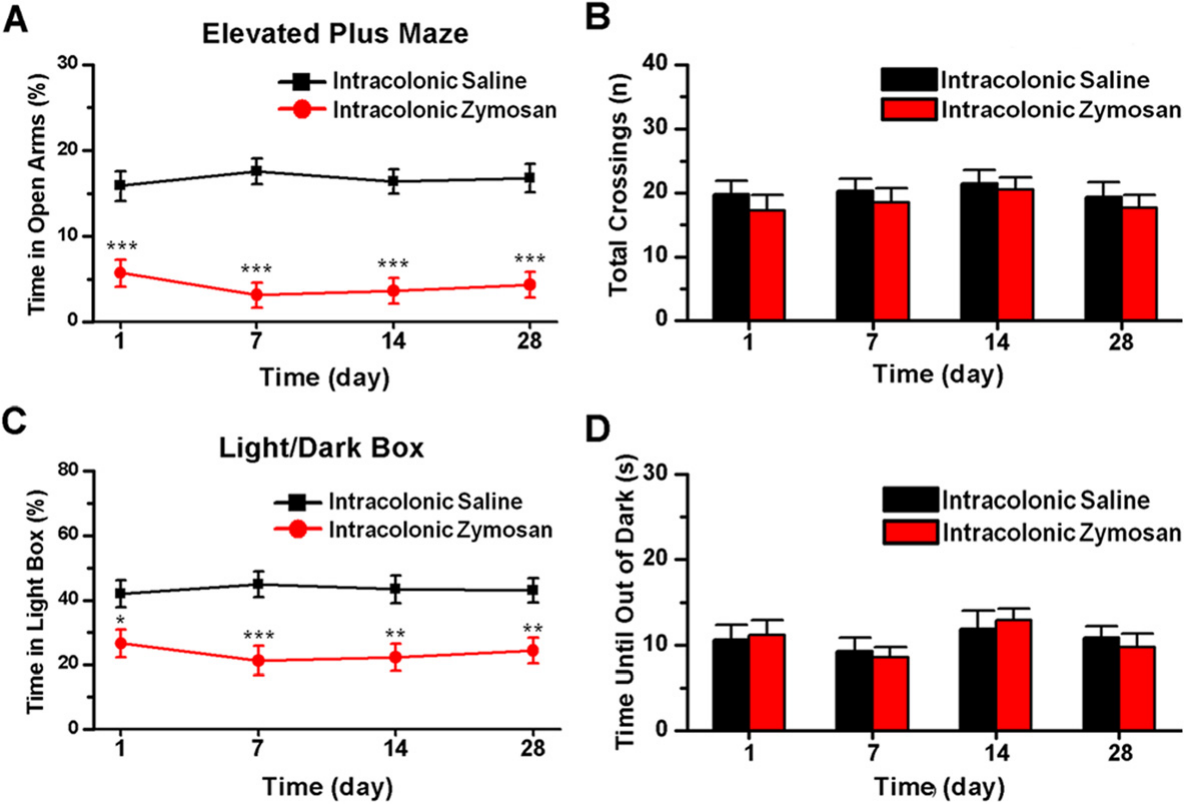
**Behavioral assessment of anxiety in zymosan-treated mice. (A)** Zymosan-treated mice exhibited significant increases in anxiety-like behaviors in the elevated plus maze compared to saline-treated mice on days 1, 7, 14 and 28 (n = 6 mice per group for each testing day). * denotes a significant difference between zymosan- and saline-treated mice. **(B)** No statistically significant difference was observed in the numbers of total crossings (open + closed) of the elevated plus maze between zymosan- and saline-treated mice on testing days (*P* = 0.25). **(C)** Zymosan-treated mice exhibited significant increases in anxiety-like behaviors in the light/dark box compared to saline-treated mice on days 1, 7, 14 and 28 (n = 6 mice per group for each testing day). * denotes a significant difference between zymosan- and saline-treated mice. **(D)** No statistically significant difference between zymosan- and saline-treated mice was observed in the time until mice exited the dark box for the first time in the light/dark box test (*P* = 0.89).

To further evaluate changes in anxiety-related behaviors, we used the elevated plus maze and a light/dark box tests. The elevated plus maze is based on an anxiogenic agent, such as an unprotected elevated area, the anxiety level being expressed by the number of entries into and the length of time spent in the aversive area [51]. The light/dark box is based on the innate aversion of rodents to brightly illuminated areas and on the spontaneous exploratory behavior of rodents in response to stress or anxiety [52]. Firstly, we found that the time spent in the open arms of the elevated plus maze was significantly reduced in mice in the day 1 group compared to saline-treated mice (*F*_(1, 40)_ = 22.63, *P* < 0.001; n = 6 mice per group for each testing day, two-way ANOVA followed by *post hoc* comparison with LSD test; Figure 4A), suggesting that zymosan treatment can induce anxiety in mice. To determine if the anxiety is long-lasting, we measured responses at other time points. We found that zymosan-treated mice spent less time in the open arms of the elevated plus maze on days 7 (*F*_(1, 40)_ = 47.09, *P* < 0.001), 14 (*F*_(1, 40)_ = 35.12, *P* < 0.001) and 28 (*F*_(1, 40)_ = 35.12, *P* < 0.001) (n = 6 mice per group for each testing day, two-way ANOVA followed by *post hoc* comparison with LSD test; Figure 4A). There was no difference among the day 1, 7, 14 and 28 groups (*F*_(3, 40)_ = 0.74, *P* = 0.53; two-way ANOVA; Figure 4A). The data suggest that zymosan-treated mice exhibit long-term anxiety that lasted at least 4 weeks. By contrast, there was no difference in the number of total crossings between zymosan-treated mice and saline-treated mice (*F*_(1,40)_ = 1.36, *P* = 0.25; two-way ANOVA; Figure 4B), indicating that the decreased activity of the zymosan-treated mice in the open arms is not due to hypoactivity.

Finally, we examined behavioral responses in the light/dark box test. We found that the time spent in the light box was markedly decreased in mice in the day 1 group compared to saline-treated mice (*F*_(1, 40)_ = 6.03, *P* < 0.05; n = 6 mice per group for each testing day, two-way ANOVA followed by *post hoc* comparison with LSD test; Figure 4C). We also examined mice at different time points using the light/dark box to evaluate the time course of zymosan-induced anxiety-like behaviors. Zymosan-treated mice spent less time in the light box on days 7 (*F*_(1, 40)_ = 15.83, *P* < 0.001), 14 (*F*_(1, 40)_ = 12.44, *P* < 0.01) and 28 (*F*_(1, 40)_ = 9.81, *P* < 0.01) (n = 6 mice per group for each testing day, two-way ANOVA followed by *post hoc* comparison with LSD test; Figure 4C). There were no significant differences among the day 1, 7, 14 and 28 groups (*F*_(3, 40)_ = 0.42, *P* = 0.74; two-way ANOVA; Figure 4C). As in the elevated plus maze, zymosan-treated mice also exhibited anxiety-like behaviors in the light/dark box, and these behaviors lasted at least 4 weeks. There were no significant differences in the time at which animals left the dark box among these groups (*F*_(1, 40)_ = 0.02, *P* = 0.89; two-way ANOVA; Figure 4D), indicating that intracolonic injection of zymosan does not influence the conflict between rodents’ exploratory tendencies and aversive properties of the environment.

### NB001 and gabapentin alleviated zymosan-induced visceral pain

Our previous studies indicated that AC1 contributes to chronic pain–related neuronal plasticity in the brain and the spinal cord [18,19,33,34,53,54]. Pharmacological inhibition of AC1 by NB001 has analgesic effects in animal models of neuropathic pain and inflammatory pain [37]. However, the effect of NB001 on IBS has not been investigated. Gabapentin is a commonly used anticonvulsant that also has analgesic effects on visceral hyperalgesia [42-44,55,56]. Therefore, we decided to investigate the effects of NB001 and gabapentin on visceral pain in zymosan-treated mice. To avoid continuous effects and interaction of drugs, independent groups were used for all testing days. NB001 (3 mg/kg) or gabapentin (30 mg/kg) was intraperitoneally (IP) injected into zymosan-treated mice 45 min prior to behavioral testing.

**Figure 5:**
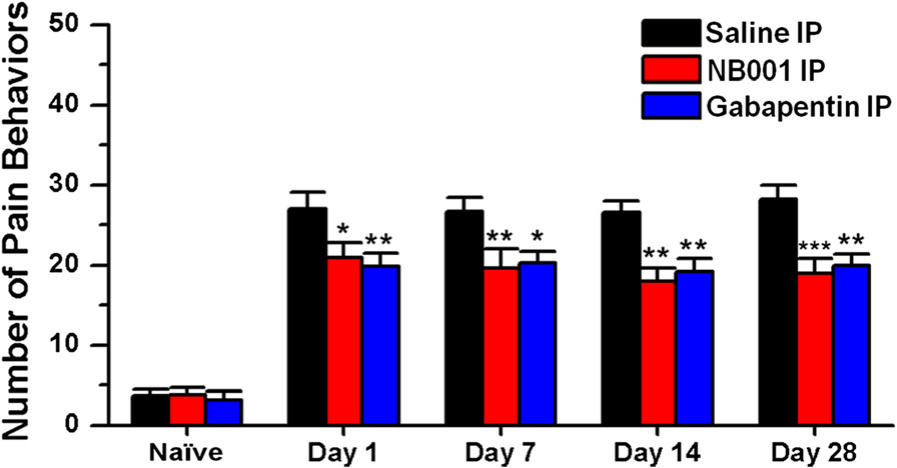
**Effects of NB001 and gabapentin on visceral pain behaviors in zymosan-treated and naïve mice.** The histogram shows that the number of visceral pain behaviors observed in zymosan-treated mice on days 1, 7, 14 and 28 decreased significantly after injection with NB001 (3 mg/kg, n = 6 mice) or gabapentin (30 mg/kg, n = 6 mice) compared to mice injected with saline (n = 6 mice). * denotes a significant difference compared to saline*-*injected mice. No significant difference was observed in the number of visceral pain behaviors among naïve mice administered NB001, gabapentin or saline (n = 6 mice per group; *P* = 0.87). Visceral pain was measured 45 min after IP injection.

We found that NB001 reduced visceral pain behaviors in animals at 1 day after zymosan treatment (NB001: 21.0 ± 1.8, saline: 27.0 ± 2.0, *P* < 0.05; n = 6 mice per group for each testing day, two-way ANOVA followed by *post hoc* comparison with Dunnett’s test; Figure 5). The inhibitory effects of NB001 were also observed at other time points (day 7, NB001: 19.7 ± 2.4, saline: 26.7 ± 1.8, *P* < 0.01; day 14, NB001: 18.0 ± 1.6, saline: 26.5 ± 1.4, *P* < 0.01; day 28, NB001: 19.0 ± 1.8, saline: 28.2 ± 1.8, *P* < 0.001; n = 6 mice per group for each testing day, two-way ANOVA followed by *post hoc* comparison with Dunnett’s test; Figure 5). There were no significant differences among the effects of NB001 within each testing days on zymosan-induced visceral pain (*F*_(3, 20)_ = 0.43, *P* = 0.73; one-way ANOVA; Figure 5). We also estimated the maximum possible inhibition (MPI) of NB001 (see Methods). The mean MPI of NB001 on visceral pain behaviors was about 30.5%.

We next examined the effects of gabapentin on visceral pain in zymosan-treated mice. We found that gabapentin injection also produced reduction in visceral pain behaviors (day 1: 19.8 ± 1.7, *P* < 0.01; day 7: 20.3 ± 1.4, *P* < 0.05; day 14: 19.2 ± 1.7, *P* < 0.01; day 28: 20.0 ± 1.4, *P* < 0.01; in comparison with saline injection group, n = 6 mice per group for each testing day, two-way ANOVA followed by *post hoc* comparison with Dunnett’s test; Figure 5). There were no significant differences among the effects of gabapentin within each testing days on zymosan-induced visceral pain (*F*_(3, 20)_ = 0.09, *P* = 0.96; one-way ANOVA; Figure 5). The mean MPI of gabapentin on visceral pain behaviors was 28.6%.

### NB001 and gabapentin alleviated zymosan-induced spontaneous pain

**Figure 6:**
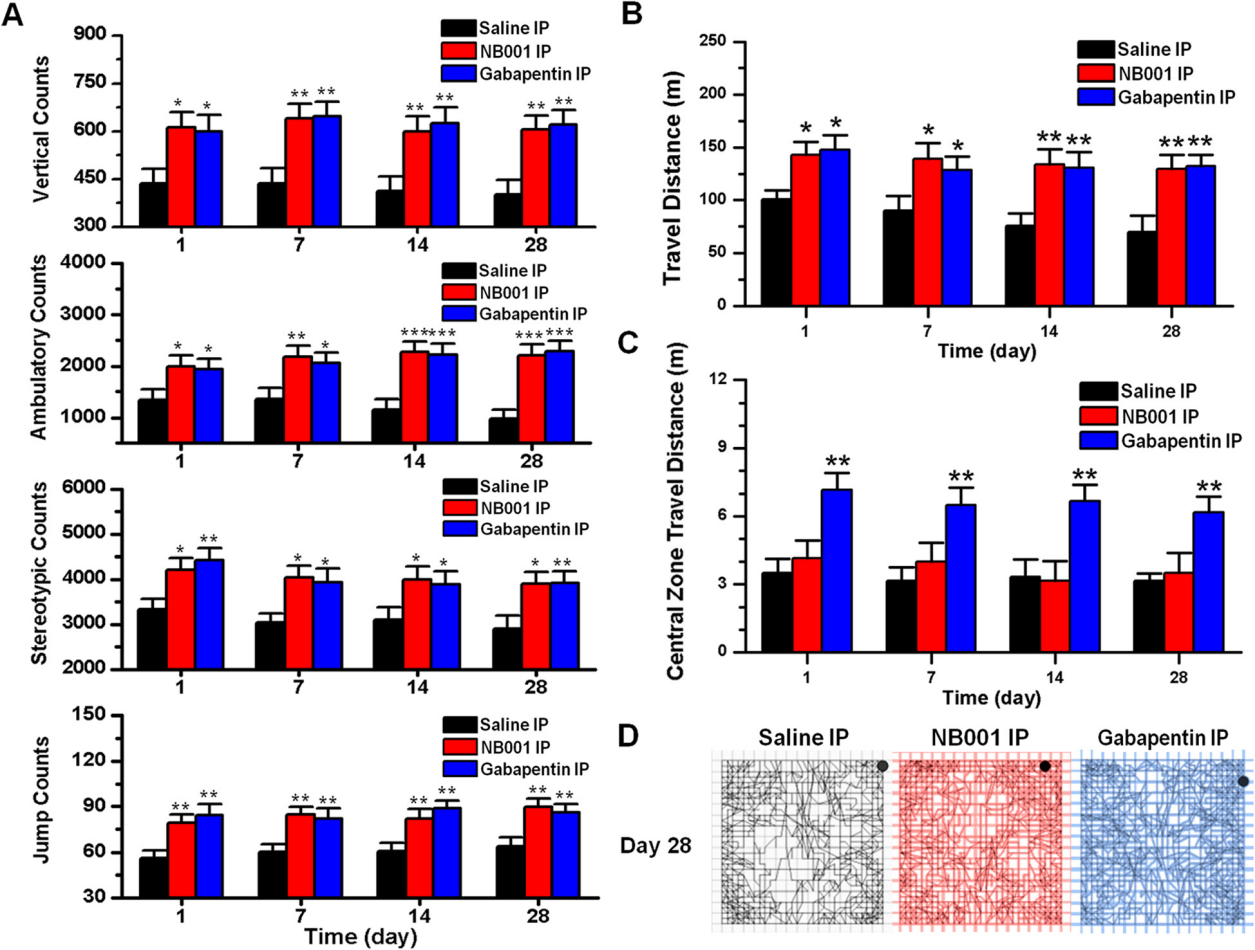
**Effects of NB001 and gabapentin on spontaneous pain and anxiety-like behaviors in the open field. (A)** Counts of vertical, ambulatory, stereotypic and jump behaviors in zymosan-treated mice on days 1, 7, 14 and 28 increased after injection of NB001 or gabapentin compared to saline injection (n = 6 mice per group for each testing day). * denotes a significant difference compared to saline*-*injected mice. **(B)** The distance traveled by zymosan-treated mice on days 1, 7, 14 and 28 increased after injection of NB001 or gabapentin compared to saline injection (n = 6 mice per group for each testing day). * denotes a significant difference compared to saline*-*injected mice. **(C)** Gabapentin injection significantly increased the distance traveled in the central zone within the first 5 min of the 30-min test session in zymosan-treated mice on days 1, 7, 14 and 28 compared to saline injection (n = 6 mice per group for each testing day). * denotes a significant difference compared to saline*-*injected mice. No significant difference was observed between zymosan-treated mice injected with NB001 or saline on testing days (*P* = 0.63). **(D)** Representative traces showed the movements of zymosan-treated mice injected with NB001, gabapentin or saline in the open field over a 30 min testing period on day 28. In each experiment, behavioral testing began 45 min after IP injection.

To evaluate further the effects of NB001 and gabapentin, the open field test was used to measure spontaneous pain. NB001 injection into zymosan-treated mice 45 min prior to open field test increased spontaneous activities on days 1, 7, 14 and 28 (n = 6 mice per group for each testing day, two-way ANOVA followed by *post hoc* comparison with Dunnett’s test; Table 2, Figure 6A). Interestingly, NB001 produced more than 50% MPI on animal’s ambulatory counts, particularly on days 1 and 14 (day 1: 103.7%; day 7: 81.6%; day 14: 94.7%; day 28: 79.9%). By contrast, the mean MPIs of NB001 on the other spontaneous activities counts were less than 40% (vertical: 30.3%; stereotypic: 39.2%; jump: 34.5%). Thus, the ambulatory counts may be the most sensitive parameter to predict the analgesic effects of drugs on spontaneous visceral pain.

**Table 2:** Effects of NB001 or gabapentin IP injection on spontaneous activities counts of zymosan-treated mice

**Counts**	**Day**	**Saline IP**	**NB001 IP**	***P***	**Gabapentin IP**	***P***
Vertical	1	437.7 ± 44.4	612.3 ± 46.7	< 0.05	599.3 ± 50.4	< 0.05
	7	437.3 ± 46.1	639.5 ± 44.8	< 0.01	646.2 ± 45.7	< 0.01
	14	411.2 ± 46.6	600.2 ± 46.4	< 0.01	623.5 ± 51.8	< 0.01
	28	400.3 ± 46.7	605.0 ± 43.9	< 0.01	621.0 ± 44.9	< 0.01
Ambulatory	1	1342.5 ± 197.6	2000.8 ± 199.2	< 0.05	1942.0 ± 193.9	< 0.05
	7	1345.5 ± 224.19	2184.5 ± 214.7	< 0.01	2058.3 ± 205.0	< 0.05
	14	1155.2 ± 211	2274.2 ± 208.5	< 0.001	2222.8 ± 212.1	< 0.001
	28	985.3 ± 172.9	2210.8 ± 206.1	< 0.001	2289.8 ± 205.0	< 0.001
Stereotypic	1	3323.5 ± 252.9,	4214.0 ± 254.7	< 0.05	4418.0 ± 272.7	< 0.01
	7	3039.7 ± 205.2	4038.2 ± 271.3	< 0.05	3944.3 ± 306.2	< 0.05
	14	3103.7 ± 264.8	4002.7 ± 280.8	< 0.05	3885.7 ± 280.8	< 0.05
	28	2895.2 ± 296.9	3904.0 ± 253.4	< 0.05	3920.2 ± 258.4	< 0.01
Jump	1	55.7 ± 5.3	79.0 ± 5.7	< 0.01	84.2 ± 7.2	< 0.01
	7	60.3 ± 4.9	84.8 ± 4.7	< 0.01	82.0 ± 6.9	< 0.01
	14	60.5 ± 5.61	82.2 ± 5.8	< 0.01	88.8 ± 5.2	< 0.01
	28	63.8 ± 5.7	89.7 ± 5.4	< 0.01	85.8 ± 5.6	< 0.01

*P*: in comparison with saline IP injection group; n = 6 mice per group.

Injection of gabapentin in zymosan-treated mice also increased spontaneous activities on days 1, 7, 14 and 28 in comparison with saline injection group (n = 6 mice per group for each testing day, two-way ANOVA followed by *post hoc* comparison with Dunnett’s test; Table 2, Figure 6A). Similar to NB001, gabapentin produced more than 50% MPI on animal’s ambulatory counts, especially on day 1 and 14 (day 1: 93.5%; day 7: 69.3%; day 14: 90.4%; day 28: 86.4%). The mean MPIs of gabapentin on the other spontaneous activities counts were less than 40% (vertical: 31.3%; stereotypic: 36.9%; jump: 36.8%). The inhibitory effects produced by NB001 and gabapentin are similar at the dosage tested (Vertical: *P* = 0.80; Ambulatory: *P* = 0.79; Stereotypic: *P* = 0.99; Jump: *P* = 0.75; two-way ANOVA followed by *post hoc* comparison with LSD test; Table 2, Figure 6A).

In comparison with saline group, the decreased total travel distance in zymosan-treated mice was also relieved by either NB001 (day 1, *P* < 0.05; day 7, *P* < 0.05; day 14, *P* < 0.01; day 28, *P* < 0.01; n = 6 mice per group for each testing day, two-way ANOVA followed by *post hoc* comparison with Dunnett’s test; Figure 6B) or gabapentin (day 1, *P* < 0.05; day 7, *P* < 0.05; day 14, *P* < 0.01; day 28, *P* < 0.05; n = 6 mice per group for each testing day, two-way ANOVA followed by *post hoc* comparison with Dunnett’s test; Figure 6B). Both drugs produced more than 50% MPI on total travel distance in zymosan-treated mice, particularly on day 1 (day 1, NB001: 78.8%, gabapentin: 83.5%; day 7, NB001: 60.4%, gabapentin: 51.6%; day 14, NB001: 59.5%, gabapentin: 56.3%; day 28, NB001: 59.2%, gabapentin: 61.7%).

### Gabapentin but not NB001 reduced anxiety-like behaviors in zymosan-treated mice

In the open field, injection of gabapentin (day 1, *P* < 0.01; day 7, *P* < 0.01; day 14, *P* < 0.01; day 28, *P* < 0.01; in comparison with saline injection group, n = 6 mice per group for each testing day, two-way ANOVA followed by *post hoc* comparison with Dunnett’s test; Figure 6C), but not NB001(day 1, *P* = 0.52; day 7, *P* = 0.42; day 14, *P* = 0.87; day 28, *P* = 0.75; in comparison with saline injection group, n = 6 mice per group for each testing day, two-way ANOVA followed by *post hoc* comparison with Dunnett’s test; Figure 6C), increased the travel distance within the central zone during the first 5 min of testing on all testing days. We also found that injection of gabapentin (day 1, *P* < 0.01; day 7, *P* < 0.001; day 14, *P* < 0.001; day 28, *P* < 0.001; in comparison with saline injection group, n = 6 mice per group for each testing day, two-way ANOVA followed by *post hoc* comparison with Dunnett’s test), but not NB001 (day 1, *P* = 0.37; day 7, *P* = 0.53; day 14, *P* = 0.61; day 28, *P* = 0.48; in comparison with saline injection group, n = 6 mice per group for each testing day, two-way ANOVA followed by *post hoc* comparison with Dunnett’s test), increased the time spent in the central zone during the first 5 min of testing on all testing days. Representative traces showed that only gabapentin*-*injected mice were more willing to explore the central zone (Figure 6D). The mean MPI of gabapentin on the central zone travelling was 96.6%, on the time spent in the central zone was 87.4%.

**Figure 7:**
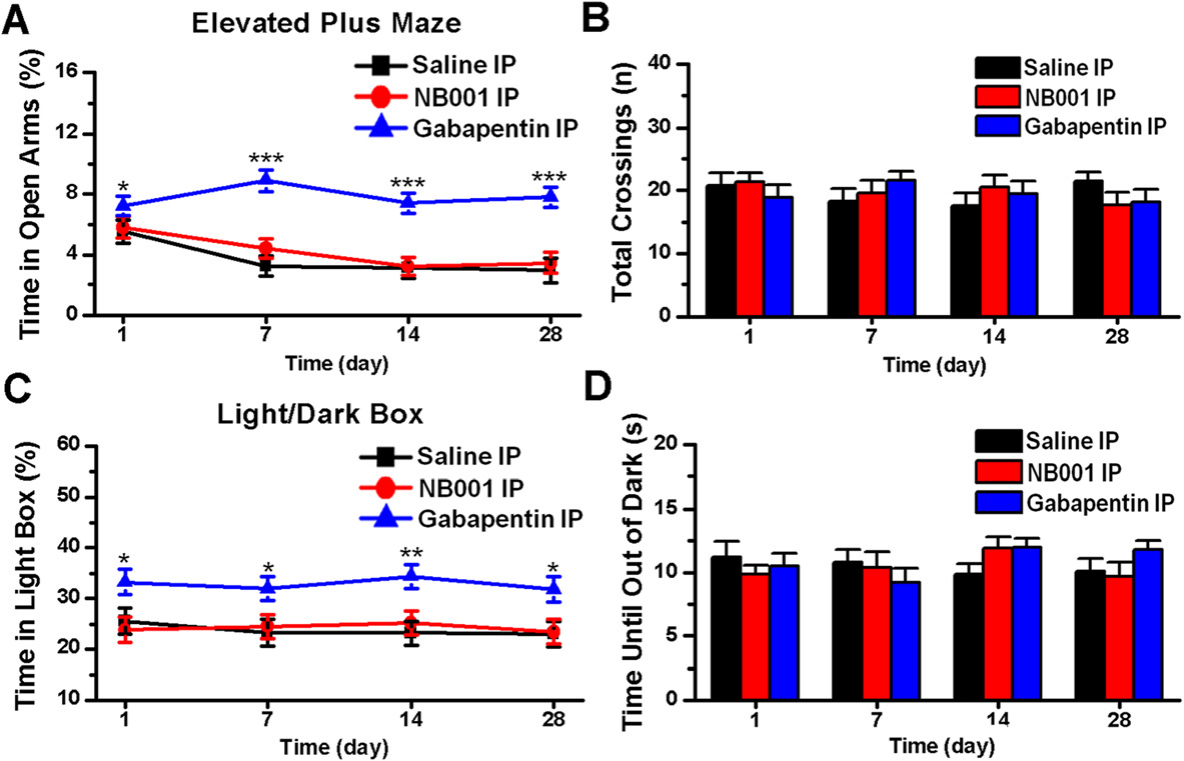
**Effects of NB001 and gabapentin on zymosan-induced anxiety-like behaviors. (A)** In the elevated plus maze, zymosan-treated mice administered gabapentin exhibited a significant reduction in anxiety-like behaviors on days 1, 7, 14 and 28 compared to mice administered saline (n = 6 mice per group for each testing day). * denotes a significant difference compared to saline*-*injected mice). No statistically significant difference was observed between zymosan-treated mice administered NB001 or saline on testing days (*P* = 0.43). **(B)** No statistically significant difference was observed in the total number of crossings (open + closed) in the elevated plus maze among zymosan-treated mice administered saline, gabapentin or NB001 on testing days (*P* = 0.80). **(C)** In the light/dark box test, zymosan-treated mice administered gabapentin exhibited a significant reduction in anxiety-like behaviors on days 1, 7, 14 and 28 compared to saline-injected mice (n = 6 mice per group for each testing day). * denotes a significant difference compared to saline-injected mice). There was no significant difference between zymosan-treated mice administered NB001 (n = 6 mice) or saline on testing days (*P* = 0.97). **(D)** No statistically significant difference was observed in the time until animals leave the dark box in the light/dark box test among zymosan-treated mice administered either saline, gabapentin or NB001 on testing days (*P* = 0.55). In each experiment, behaviors were measured 45 min after IP injection.

Mice injected with gabapentin spent more time in the open arms of the elevated plus maze (day 1, *P* < 0.05; day 7, *P* < 0.001; day 14, *P* < 0.001; day 28, *P* < 0.001; in comparison with saline injection group, n = 6 mice per group for each testing day, two-way ANOVA followed by *post hoc* comparison with Dunnett’s test; Figure 7A). The anti-anxiety effects of gabapentin in the elevated plus maze were similar within each testing days (*F*_(3, 20)_ = 1.23, *P* = 0.33; one-way ANOVA; Figure 7A). The averaged MPI of gabapentin on zymosan-treated mice in the elevated plus maze was 28.4%. However, NB001 had no obvious effect on it (day 1, *P* = 0.73; day 7, *P* = 0.73; day 14, *P* = 0.99; day 28, *P* = 0.61; in comparison with saline injection group, n = 6 mice per group for each testing day, two-way ANOVA followed by *post hoc* comparison with Dunnett’s test; Figure 7A). Neither drug influenced the total number of crossings in the elevated plus maze at each time point (*F*_(3, 60)_ = 0.34, *P* = 0.80; two-way ANOVA; Figure 7B).

Finally, to further confirm the effect of gabapentin, we performed experiments using the light/dark box. Mice treated with gabapentin (day 1, *P* < 0.05; day 7, *P* < 0.05; day 14, *P* < 0.01; day 28, *P* < 0.05; in comparison with saline injection group, n = 6 mice per group for each testing day, two-way ANOVA followed by *post hoc* comparison with Dunnett’s test; Figure 7C), but not NB001 (day 1, *P* = 0.63; day 7, *P* = 0.85; day 14, *P* = 0.57; day 28, *P* = 0.89; in comparison with saline injection group, n = 6 mice per group for each testing day, two-way ANOVA followed by *post hoc* comparison with Dunnett’s test; Figure 7C), spent more time in the light box. Neither drug influenced the time at which animals left the dark box at each time point (*F*_(3, 60)_ = 0.71, *P* = 0.55; two-way ANOVA; Figure 7D), indicating that neither NB001 nor gabapentin influences the conflict between rodents’ exploratory tendencies and aversive properties of the environment. Gabapentin produced more than 40% MPI on zymosan-treated mice in the light/dark box, particularly on day 14 (day 1: 49.4%; day 7: 45.4%; day 14: 59.2%; day 28: 40.5%). The data reveal that gabapentin alleviates anxiety-like behaviors via a mechanism unrelated to antinociception, whereas NB001 is unable to alleviate zymosan-induced anxiety-like behaviors in mice.

## Discussion

In the present study, we examined the neurobehavioral manifestations of zymosan-induced colitis in mice and the effects of NB001 and gabapentin on mice treated with zymosan. Our results included three novel findings: (1) Elevated expression of Fos protein in the brains of zymosan-treated mice demonstrated that cortical areas are involved in the regulation of pain and emotions in mice experiencing zymosan-induced colitis. (2) Zymosan-treated mice exhibited obvious, spontaneous visceral pain and anxiety-like behaviors, which persisted for at least 4 weeks after treatment. (3) An investigation of the effects of NB001 and gabapentin on zymosan-induced colitis showed that both drugs had markedly antinociceptive effects. Furthermore, gabapentin, but not NB001, was able to alleviate anxiety in zymosan-treated mice.

### Fos protein was expressed at high levels in the brain after the induction of colitis with zymosan

Analysis of serial coronal sections showed elevated levels of Fos protein in the PFC, ACC, insular cortex, amygdala, PAG, PBN and nucleus of solitary tract in zymosan-treated mice. Among these brain regions, the PFC, ACC, insular cortex, amygdala and PAG are involved in the processing of pain information and emotional responses [18], the lateral region of PBN is involved in the processing of general visceral inputs [57], and the medial and commissural parts of the nucleus of solitary tract are the primary targets for afferent vagal fibers [58]. Our observations demonstrate that zymosan-induced colitis causes obvious nociception in mice. The elevated levels of Fos protein in some brain areas related to emotional processing suggests that mice may suffer from emotional disorders following the induction of colitis with zymosan.

### Intracolonic injection of zymosan induced chronic visceral pain

Previous work showed that intracolonic treatment with zymosan could produce a model of chronic colon hypersensitivity involving a transient low-level inflammation [40,41,47]. Thus, zymosan is capable of producing visceral hypersensitivity in rodents. Zymosan induced visceral pain is a suitable model to study potential mechanisms underlying visceral hypersensitivity, including human IBS, because of its lack of associated colon pathology. In the present study, typical chronic visceral pain behaviors, such as licking of the abdomen in the absence of other grooming behavior, whole-body stretching, flattening the abdomen against the floor and contracting the abdominal wall, were observed in zymosan-treated mice, in agreement with prior studies. We also observed long-lasting reductions in ambulatory, vertical, stereotypic and jump counts, as well as travel distance, in zymosan-treated mice when tested in the open field. These findings suggest that the abdominal visceral pain caused by zymosan influences the spontaneous responses and action intention of mice. A similar phenomenon of reduced activity occurs in people suffering from IBS, as well [59,60]. Thus, spontaneous visceral pain induced by intracolonic injection of zymosan could be measured via decreased spontaneous activity in the open field. Moreover, measurement of spontaneous activities in the open field test could be used to predict the ability of experimental IBS therapies to reduce spontaneous visceral pain.

### Zymosan-treated mice exhibited persistent anxiety-like behaviors

There is a high rate of co-morbidity between IBS and psychological disorders such as anxiety, stress and depression [11-15]. We demonstrated that zymosan-treated mice experienced more anxiety compared to saline-treated mice, as measured by the distance traveled and time spent in the central zone in the open field test and using the elevated plus maze and the light/dark box tests. Thus, visceral noxious stimulation is interpreted to cause interoceptive anxiety in zymosan-treated mice. Previous human and animal studies also showed that anxiety-like behaviors could be induced by inflammatory pain, chronic musculoskeletal pain or neuropathic pain [61-67]; but see [68]. In addition, recent research suggests that dysbiosis of the gut microbiota is implicated in the pathogenesis of IBS [69]. Commensal bacteria are known to affect a variety of complex behaviors, including social, emotional, and anxiety-like behaviors, and contribute to brain development and function in mice and human [70,71]. Thus, intracolonic treatment with zymosan may cause intestinal dysbiosis and lead to anxiety.

### Effects of NB001 on zymosan-treated mice

AC1 is activated by calcium-calmodulin- (CaM-) dependent manner. It acts downstream from glutamate NMDA receptors and contributes to chronic pain-related neuronal plasticity in the cortex and spinal cord [18,19,33,34,53,54]. In the present study, NB001, an AC1 inhibitor, exerted obvious antinociceptive effects on visceral pain induced by intracolonic injection of zymosan. Because NB001 has no effect on motor function in normal mice [37], we speculate that NB001 increases the spontaneous behaviors of zymosan-treated mice by attenuating spontaneous visceral pain. Our data suggest that the AC1 signaling pathway plays an important role in the processing of visceral pain induced by intracolonic injection of zymosan. According to the MPI, the ambulatory counts may be the most sensitive parameter to predict the analgesic effects of drugs on spontaneous visceral pain.

NB001 did not dampen anxiety-like behaviors in zymosan-treated mice, consistent with our previous finding that NB001 had no effect on anxiety-like behaviors in normal mice [37]. We believe that pain causes cortical plasticity and that brains likely exhibit ‘metaplasticity’ in response to continuous inputs from the periphery [18,72]. Thus, persistent, noxious visceral stimuli in the present study were able to cause a long-lasting pain-related emotional disorder, in this case anxiety. In addition, although pain-related anxiety is coupled to pain, growing evidence indicates that pain-related anxiety is dissociated from pain perception [73]; this finding provides an explanation for our observations that NB001 is unable to alleviate zymosan-induced anxiety by attenuating visceral pain in mice and that gabapentin can reduce anxious behaviors by a mechanism unrelated to antinociception. More research is needed to find additional methods for attenuating pain-induced anxiety.

### Effects of gabapentin on zymosan-treated mice

Gabapentin interacts with a subunit of voltage-sensitive calcium (Ca^2+^) channels [74] and modulates the function of peripheral and central pain pathways by influencing fast synaptic transmission and neuronal excitability [75,76]. Consistent with previous work, our data suggest that gabapentin relieves chronic visceral pain induced by intracolonic injection of zymosan and reduces spontaneous pain through a multiplicity of mechanisms. In addition, gabapentin has also been associated with a reversal of anxiety-like behaviors in animal models of neuropathic pain [77,78]. The present results indicated that gabapentin significantly decreases anxiety-like behaviors in zymosan-treated mice, which is coincident with its anti-nociceptive effect. Our observation that gabapentin reduces anxiety-like behaviors in zymosan-treated mice provides evidence to support the potential use of gabapentin for the treatment of IBS-related emotional disorders. However, the side effects of gabapentin, including hepatotoxicity and neurologic toxicities [79-81], should be taken into account if this drug is considered for the symptomatic treatment of IBS.

For the first time, we have presented evidence that changes in spontaneous behavioral responses are a reflection of spontaneous pain induced by intracolonic injection of zymosan. Mice with zymosan-induced colitis exhibited significant increases in anxiety-like behaviors. We first demonstrated that the expression of Fos protein is notably increased in several brain areas, and particularly in regions involved in the processing of pain and emotions; this finding suggests that these cortical and subcortical areas are involved in the response to zymosan-induced colitis. From the point of view of translational medicine, we found that IP injection of NB001 relieved spontaneous visceral pain induced by intracolonic injection of zymosan but had no effect on anxiety, whereas IP injection of gabapentin alleviated both spontaneous visceral pain and anxiety in zymosan-treated mice. Our findings provide a basis for these drugs to be considered for the clinical pharmacological treatment of IBS.

## Methods

### Animals

Experiments were performed with adult (age 8–12 weeks) male C57/BL6 mice purchased from Charles River Laboratories (St. Constant, Quebec, Canada). Animals were housed under standard laboratory conditions (12 h light/12 h dark, temperature 22-26°C, humidity 55-60%) with water and mice chow available *ad libitum*. All experiments were performed in accordance with protocols approved by the Animal Care Committee of the University of Toronto and Animal Care and Use Committee of the Fourth Military Medical University.

### Zymosan-induced colitis in animals

Colitis was induced in animals by the administration of zymosan, as described previously [41,47,48]. Mice were anesthetized by isoflurane inhalation (1-3%, or as needed). During surgery, body temperature was maintained at 37°C using a heating pad. A lubricant ointment (artificial tears) was applied to the eyes. A volume of 0.1 ml zymosan suspension (30 mg/ml in saline; derived from Saccharomyces cerevisiae; Sigma, St. Louis, MO) was administered transanally via a 22-gauge, 24-mm-long stainless-steel feeding needle into the colons of mice over a period of 2 min. A control group of mice was subjected to a similar procedure except that 0.1 ml saline was administered transanally. Zymosan or saline were given daily for three consecutive days.

### Immunohistochemical staining

On day 7, zymosan-treated mice and saline-treated mice were deeply anesthetized and perfused with 50 ml of 0.9% saline, followed by 100 ml of 0.1 M PB containing 4% (w/v) paraformaldehyde. Immediately after perfusion, brains were removed and placed into 0.1 M PB containing 30% (w/v) sucrose overnight at 4°C and cut into 25 μm-thick serial frontal sections on a freezing microtome (Kryostat 1720; Leitz, Mannheim, Germany). The sections were collected in sequence, divided into 2 series and washed with 0.01 M phosphate buffer solution (PBS, pH 7.4). The sections were incubated with mouse anti-Fos antiserum (1:500; Abcam, Cambridge, MA, USA) in PBS containing 5% (v/v) normal donkey serum (NDS), 0.3% (v/v) Triton X-100, 0.05% (w/v) NaN_3_ and 0.25% (w/v) carrageenan (PBS-NDS, pH 7.4) overnight at 4°C.

Next, the sections were washed and then incubated with biotinylated anti-mouse IgG (1:200, Millipore Corporation, USA) for 2 h at room temperature (approximately 22°C). Sections were then washed (3 × 10 min) in PBS and incubated in avidin-biotin complex (ABC kit, Vector Laboratories, Inc., USA) for 30 min. After incubation in avidin-biotin complex, sections were washed (3 × 10 min) again and reacted with 0.05 M of Tris–HCl buffer (pH 7.6) containing 0.04% diaminobenzidinetetrahydrochloride (DAB) (Dojin, Kumamoto, Japan) and 0.003% H_2_O_2_ for visualizing Fos; Fos-positive nuclei appear dark brown. Sections were washed again (once immediately after removing the DAB solution, then 3 × 10 min), then mounted on gelatin-coated glass slides, and allowed to air dry overnight. Sections were dehydrated in increasing concentrations of ethanol, cleared, and coverslipped for examination by light microscopy (AHBT3; Olympus, Tokyo, Japan).

### Quantification of Fos expression

The counts of Fos-ir neurons in the brain regions were represented as the average number of neurons per section. We limited our analysis to the PFC, ACC, insular cortex, amygdala, PAG, NTS, and PBN because most immunolabeled neurons in the brains of zymosan-treated mice were in these regions. The numbers of labeled neurons in a given brain region were estimated from the counts of positively stained cells in a minimum of 6 sections per brain region. To avoid counting a neuron more than once, sections used for the counts were separated by at least 100 μm. The differences in cell counts were evaluated statistically by unpaired *t*-test to examine the interactions among groups. Counting was performed by an investigator who was blind to the treatment condition.

### Behavioral testing

Counts of visceral pain behaviors: Visceral pain behaviors, as described by Laird [49], included licking of the abdomen in the absence of other grooming behavior, whole-body stretching, flattening the abdomen against the floor, or contracting the abdominal wall such that an arched posture was adopted for 1–2 sec (abdominal retractions). The total number of visceral pain behaviors was recorded over a period of 10 min.

Open field: Mice were placed in a novel open field (43.2 × 43.2 × 30.5 cm^3^; Med Associates, St. Albans, Vermont) inside a dimly lit isolation chamber (<50 lux in the center of the open field) with a fan. An activity monitoring system (Activity Monitor, Med Associates, St. Albans, Vermont) was used to record horizontal locomotor activity. Briefly, this system uses paired sets of photo beams to detect movement (number of photo beams: 16; space between the beams: 2.5 cm; number of zones: X: 17, Y: 17). Each animal was placed in the center of the open field, and activity was measured for 30 min. Central zone was defined by zones from (4, 4) to (13, 13).

Elevated plus maze: The elevated plus maze (Med Associates, St. Albans, Vermont) consisted of two open arms in line with each other placed perpendicularly to two closed arms in line with each other [82]. The maze was situated 70 cm from the floor. For each test, individual animals were placed in the center square with the head facing a closed arm. The number of entries into and time spent in each arm were recorded for 5 min.

Light/dark box: The light/dark box used in this study was a modified version of a light/dark box described previously by Kim *et al.*[23]. The apparatus consisted of a rectangular Plexiglas box (44 × 8.5 × 25 cm^3^) divided into equal-size light and dark compartments separated by a door. The light box was lit by a 60-W desk lamp (400 lux) placed 30 cm above the box. Each animal was placed in the dark compartment and was allowed 20 section to explore, after which the door to the light compartment was opened. The time until the animal left the dark box and the time spent in the light box were recorded for 10 min.

Mice were acclimatized to the observation room for 30 min prior to behavioral tests. The behavioral tests were performed by an investigator who was blind to the treatment condition from 9:00 am to 12:00 pm.

### Drugs injection

Mice were administered NB001 3 mg/kg or gabapentin (Tocris Bioscience) 30 mg/kg IP prior to behavioral tests.

### Statistical analysis

All data were collected by experimenters who were blind to the surgical and chemical treatments. Statistical analysis was performed using SPSS software (version 21). Data were expressed as the mean ± standard error of the mean (mean ± S.E.M.). Inhibition of visceral pain or anxiety was presented as maximum possible inhibition (MPI) = (P_drug_ - P_zymosan_)/(P_saline_ - P_zymosan_) × 100% (P_drug_: value of parameter detected in each behavior test in zymosan-treated mice with NB001 or gabapentin IP injection; P_zymosan_: value of parameter detected in each behavior test in mice with zymosan intracolonic treatment; P_saline_: value of parameter detected in each behavior test in mice with saline intracolonic treatment). When necessary, statistical significance was assessed by unpaired *t*-test, repeated measures ANOVA followed by *post hoc* comparison with LSD test, one way ANOVA, two-way ANOVA followed by *post hoc* comparison with LSD test or two-way ANOVA followed by *post hoc* comparison with Dunnett’s test. A threshold for statistical significance of *P* < 0.05 was chosen.

## Abbreviations

AC: Adenylyl cyclase; ACC: Anterior cingulate cortex; ACSF: Artificial cerebrospinal fluid; Amy: Amygdala; cc: Central canal; CeA: Central amygdala; DAB: Diaminobenzidinetetrahydrochloride; IBS: Irritable bowel syndrome; IC: insular cortex; MPI: Maximum possible inhibition; PAG: Periaqueductal gray; PBN: Parabrachial nucleus; PBS: Phosphate buffer solution; PFC: Prefrontal cortex; NTS: Nucleus of solitary tract.

## Competing interests

The authors declare that they have no competing interests.

## Authors’ contributions

MMZ and SBL are responsible for performance of experiments and writing the manuscript. TZ and KK are responsible for collecting data. MMZ and MZ are responsible for experimental design and writing the manuscript. All authors read and approved the final manuscript.
